# Improved physiology and metabolic flux after Roux-en-Y gastric bypass is associated with temporal changes in the circulating microRNAome: a longitudinal study in humans

**DOI:** 10.1186/s40608-018-0199-z

**Published:** 2018-05-31

**Authors:** Abdullah Alkandari, Hutan Ashrafian, Thozhukat Sathyapalan, Peter Sedman, Ara Darzi, Elaine Holmes, Thanos Athanasiou, Stephen L. Atkin, Nigel J. Gooderham

**Affiliations:** 1 Department of Surgery and Cancer, Imperial College London, London, UK; 2 Clinical Trials Unit, Dasman Diabetes Institute, PO Box 1180, Dasman, 15462 Kuwait City, Kuwait; 3 Department of Bariatric and Metabolic Surgery, Chelsea and Westminster NHS Foundation Trust, London, UK; 4 Department of Academic Endocrinology, Diabetes and Metabolism, Hull York Medical School, Hull, UK; 5 Division of Upper Gastrointestinal and Minimally Invasive Surgery, Hull and East Yorkshire Hospitals NHS Trust, Hull, UK; 6 Weill Cornell Medical College Qatar, Qatar Foundation, Doha, Qatar

**Keywords:** MicroRNA, miRNA, Circulating, Bariatric, Gastric bypass, RYGB, Biomarker, Longitudinal, Temporal

## Abstract

**Background:**

The global pandemic of obesity and the metabolic syndrome are leading causes of mortality and morbidity. Bariatric surgery leads to sustained weight loss and improves obesity-associated morbidity including remission of type 2 diabetes. MicroRNAs are small, endogenous RNAs that regulate gene expression post-transcriptionally, controlling most of the human transcriptome and contributing to the regulation of systemic metabolism. This preliminary, longitudinal, repeat sampling study, in which subjects acted as their own control, aimed to assess the temporal effect of bariatric surgery on circulating microRNA expression profiles.

**Methods:**

We used Exiqon’s optimized circulating microRNA panel (comprising 179 validated miRNAs) and miRCURY locked nucleic acid plasma/serum Polymerase Chain Reaction (PCR) to assess circulating microRNA expression. The microRNAome was determined for Roux-en-Y gastric bypass (RYGB) patients examined preoperatively and at 1 month, 3 months, 6 months, 9 months and 12 months postoperatively. Data was analysed using multivariate and univariate statistics.

**Results:**

Compared to the preoperative circulating microRNA expression profile, RYGB altered the circulating microRNAome in a time dependent manner and the expression of 48 circulating microRNAs were significantly different. Importantly, these latter microRNAs are associated with pathways involved in regulation and rescue from metabolic dysfunction and correlated with BMI, the percentage of excess weight loss and fasting blood glucose levels.

**Conclusions:**

The results of this pilot study show that RYGB fundamentally alters microRNA expression in circulation with a time-dependent progressive departure in profile from the preoperative baseline and indicate that microRNAs are potentially novel biomarkers for the benefits of bariatric surgery.

**Electronic supplementary material:**

The online version of this article (10.1186/s40608-018-0199-z) contains supplementary material, which is available to authorized users.

## Introduction

Bariatric surgery has been established as the most effective strategy for inducing sustained weight loss and enhancing metabolism to manage morbid obesity and its systemic co-morbidities. These procedures improve longevity and quality of life and lead to remission of type 2 diabetes mellitus (T2DM) and reduction in cardiovascular risk, often independent of weight loss [1]. The physiological effects of bariatric surgery include the BRAVE steps (bile flow alteration, reduction of gastric size, anatomical gut rearrangement and altered flow of nutrients, vagal manipulation and enteric gut hormone modulation) with the associated modulation of the gut microbiome and a multitude of downstream physiological and disease-modifying effects [2, 3]. However, the precise mechanism behind bariatric surgery remain poorly understood.

MicroRNAs are a family of endogenous, non-coding RNAs that regulate gene expression at the post-transcriptional level. These small, evolutionarily conserved RNA transcripts bind to target messenger RNA (mRNA) transcripts leading to mRNA cleavage or translational repression [4]. This enables each of the approximately 3000 distinct human microRNA sequences to fine-tune or silence several mRNA targets involved in a diverse array of cellular pathways. Thus, microRNAs form a complex network of gene ‘super-regulators’ controlling virtually every biological process. As such microRNAs have been implicated in the progression of obesity and its many co-morbidities. MicroRNAs regulate adipogenesis, insulin secretion, glucose uptake and lipid metabolism and many other biological processes linked with obesity [5].

While microRNAs act at the subcellular level, they are also found in the circulation within exosomes or complexed with high-density lipoproteins or argonaute proteins [6]. The mechanisms whereby microRNAs appear in the circulation and the biological implications of circulation expression are not understood. However, microRNAs are stable in circulation, not readily digested by ribonucleases and able to withstand repeated freeze-thaw cycles allowing for long-term storage of circulatory fluids without compromising the microRNA integrity [7]. They can also be measured with speed, ease and sensitivity through quantitative PCR and other platforms. This has given microRNAs rich clinical and therapeutic potential as novel biomarkers [8]. Recent studies have reported circulating microRNAs as biomarkers for cancer, obesity, diabetes and many other disorders [7, 9, 10].

Recently, single post-surgical timepoint studies have shown that bariatric surgery modulates circulating microRNA expression [9, 11, 12]. The objective of the present work was to complete a longitudinal repeat sampling study in which patients were their own preoperative controls, to assess the temporal effect of bariatric surgery on circulating microRNA expression profiles and to identify potential microRNA biomarkers.

## Methods

### Recruitment

Plasma samples were collected from 4 men and 5 women undergoing laparoscopic RYGB at Hull and East Yorkshire Hospitals NHS Trust in the United Kingdom under ethics approval 10/H1304/13. All patients were morbidly obese preoperatively and met qualifying criteria for bariatric surgery set out by NICE [13]. Plasma samples were collected in a fasting state and body mass index (BMI) and fasting blood glucose were measured immediately preoperatively and where possible at 1 month, 3 months, 6 months, 9 months and 12 months postoperatively. All participants provided informed written consent prior to the study.

### Sample preparation and extraction

Plasma samples were acquired by standard venipuncture and centrifugation in EDTA-coated vacutainer tubes (Becton Dickinson) and frozen at -80 °C until use. RNA was extracted from plasma using the *mir*Vana PARIS Isolation Kit (Life Technologies), according to the manufacturer’s instructions with one modification; prior to the addition of acid-phenol:chloroform, 150Amoles of c-elegans miR 39 (Life Technologies) was added as an internal standard and RNA carrier. RNA was extracted from 100 μl plasma and eluted in 100 μl nuclease free water. A 4 μl aliquot of RNA elute was reverse transcribed in 20 μl reactions using the Universal cDNA Synthesis Kit (Exiqon), according to the manufacturer’s instructions.

### Profiling of circulating microRNA

Plasma samples were profiled using Exiqon’s miRCURY locked nucleic acid (LNA) platform and Serum/Plasma Focus microRNA PCR panels, according to the manufacturer’s instruction. Panels comprised two 96-well plates coated with LNA microRNA primers for 179 microRNAs, selected and optimized by the manufacturer for their typical expression in circulation [14]. No template and UniSp3 spike-in primers were included for quality control. PCR was carried out on the StepOnePlus 7500 PCR system (Life Technologies) using the SYBR Green dye as instructed by the manufacturer.

### Data analysis

Pre-processing and initial analysis of profiling data was performed with Exiqon’s GenEx6 software. The threshold for expression was set as Cq < 37. MicroRNAs not detected in over 60% of samples were removed from consideration. Individual microRNA expression levels are expressed relative to preoperative expression. The miRWalk (v.2) database of predicted and validated microRNA targets and the PANTHER classification system were used to allocate regulated pathways as previously described [15]. To ensure confidence and accuracy, only mRNA targets predicted by 2 or more algorithms were included in the analysis. Statistical significance was determined by ANOVA and the Student’s t test with Sidak-Bonferroni correction for multiple comparisons as appropriate. Expression of microRNAs was correlated with clinical parameters by the Pearson test. Principal component analysis (PCA) pattern recognition was applied to visualise differences in whole microRNA profiles prior to and following RYGB. All analysis was performed using GraphPad Prism 6.0 and R (www.r-project.org).

## Results

As expected, all patients demonstrated substantial time-dependent reduction in weight following surgery (Table 1). Mean BMI decreased gradually and significantly from 49 kg/m^2^ to 30.7 kg/m^2^ and patients lost on average 72% of their mean preoperative excess weight 1 year following surgery. Although there were no significant changes in mean fasting blood glucose following surgery, only 2 out of the 9 patients in this study were diabetic and both demonstrated decreases in postoperative fasting blood glucose relative to their individual preoperative level.

**Table 1:** Summary of patient information and clinical outcomes of surgery^a^

Patients	Pre	1 m	3 m	6 m	9 m	12 m	ANOVA
n	9	6	8	7	7	4	
Age	46.1 (9.8)	–	–	–	–	–	–
Sex (Female)	5/9	–	–	–	–	–	–
Diabetics	2/9	–	–	–	–	–	–
BMI (kg/m^2^)	49 (10)	41.2 (7.9)	37.5 (6.5)	36.4 (8.2)	33.7 (7.9)	30.7 (4.5)	0.0028 (**)
%EWL	0.0	33.7 (13.5)	48.2 (16.8)	55.9 (18.5)	68.9 (17.5)	71.7 (16.7)	0.0044 (**)
Fasting blood glucose (mmol/L)	7.5 (5.4)	4.2 (0)	6.9 (3.5)	5.7 (1.4)	5.6 (1.4)	5.9 (1.3)	0.733

^a^*BMI* body mass index, *EWL* excess weight lost. Parentheses indicate standard deviation. **p < 0.001 by ANOVA

### Normalization

Profiling found 159 microRNAs that surpassed detection threshold. This offered multiple normalizing options to minimize technical variation. Five different approaches were assessed; microRNA expression was (a) not normalized, (b) normalized to the mean expression of all expressed microRNAs (global mean), (c) normalized to miR 223-3p and 26a, relatively stable microRNAs as determined by geNorm [16], (d) normalized to miR 101-3p and 19a, relatively stable microRNAs as determined by Normfinder [17] and (e) normalized to a combination of these 4 endogenous microRNAs. Overall variation was assessed through coefficient of variances (CV) and cumulative distribution as previously described [18] (Additional file 1: Figure S1). The optimum method was normalization to the combination of miR 223-3p miR 26a, miR 101-3p and miR 19a which displayed significantly lower mean CVs of the 50% least variable microRNAs compared to all other assessed methods (Additional file 1: Figure S1B). As a result, all profiling data were normalized to these 4 endogenous microRNAs.

### Multivariate analysis

Using an untargeted approach, multivariate statistical analysis with PCA scores plot demonstrated separation between the preoperative and 5 postoperative groups (Additional file 2: Figure S2A). There appeared to be discrete clustering according to time: preoperative profiles (pink); 1 month postoperative profiles (red); 3 months postoperative profile (green); 6 month postoperative profiles (light blue); 9 month postoperative profiles (blue); and 12 month postoperative profiles (black). This powerful untargeted approach was further demonstrated in the trajectory PCA, which plots the mean components of all 6 groups (Additional file 2: Figure S2B). The preoperative and 1 month postoperative microRNA profiles were similar but there is a notable shift of increasing separation with each sequential postoperative timepoint. This trajectory shift is also evident in the preoperative-postoperative comparative heatmap of microRNA expression and the associated group cluster dendrogram (Additional file 2: Figure S2C and D).

### Univariate analysis

**Fig. 1:**
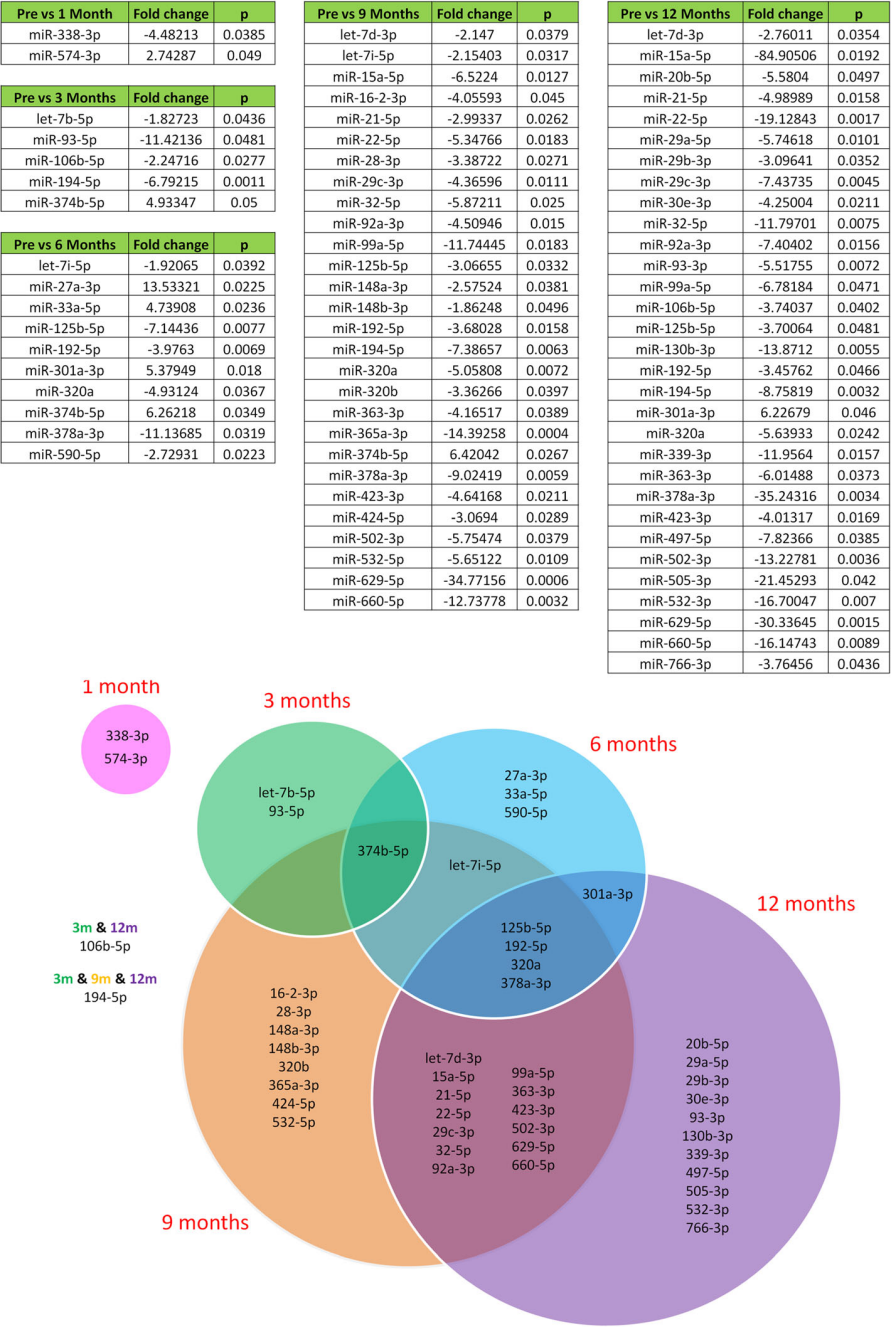
Circulating microRNAs significantly deregulated following RYGB. (Top) Circulating microRNAs significantly differentiated at 1 month, 3 months, 6 months, 9 months and 12 months following RYGB. Statistical significance was determined by Student’s t-test with Sidak-Bonferroni correction for multiple comparisons. (Bottom) A Venn diagram illustrating which significantly differentiated microRNAs are unique and shared between timepoints following RYGB

**Fig. 2:**
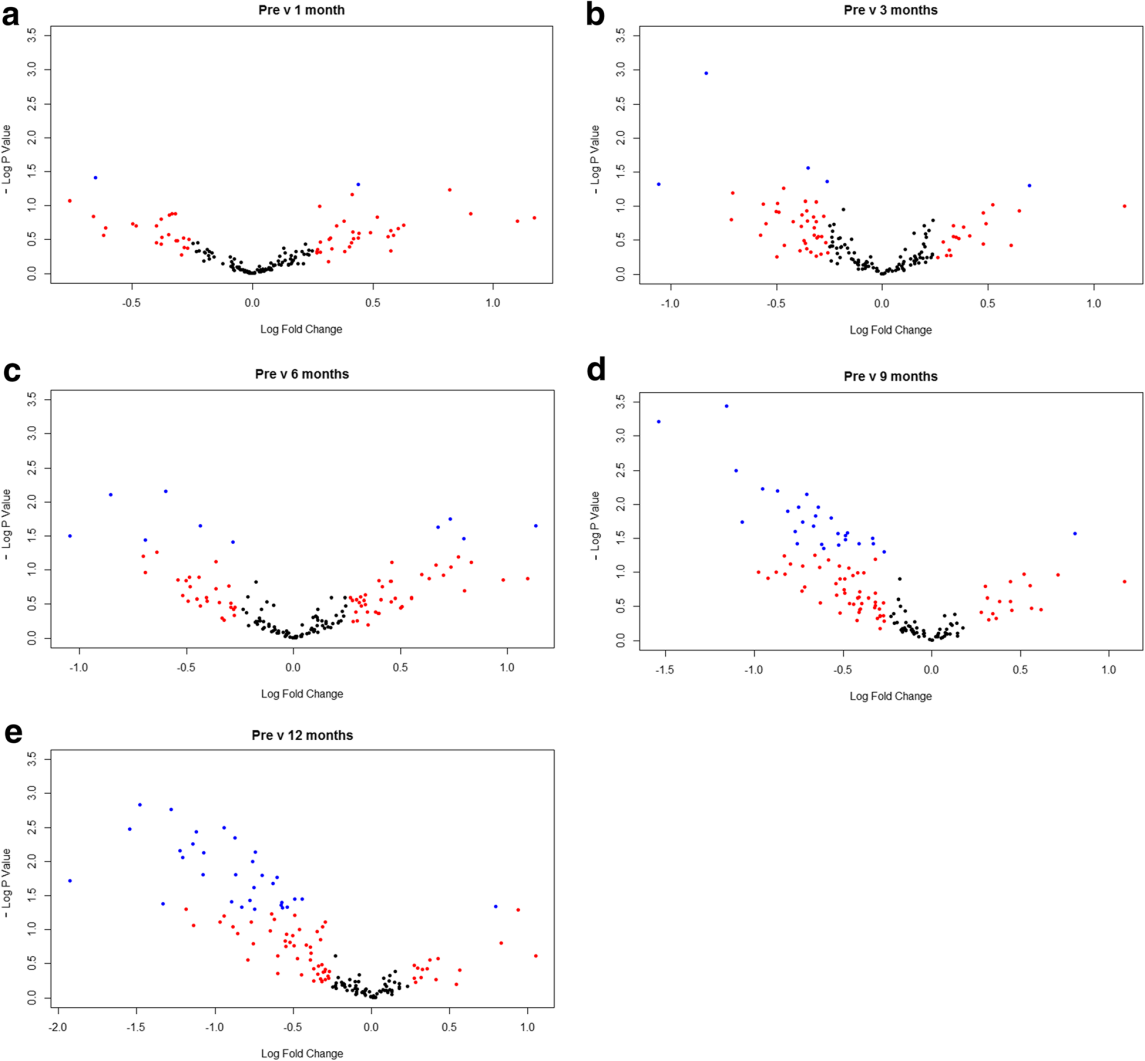
Univariate analysis of profiling data presented in volcano plots. Log fold change against -log *p* value by Student’s t test of postoperative microRNA expression at (**a**) 1 month, (**b**) 3 months, (**c**) 6 months, (**d**) 9 months and (**e**) 12 months following RYGB relative to preoperative levels. Each data point represents an individual microRNA that has passed expression threshold. Data points in blue are statistically significant (*p* < 0.05)

Since each patient served as their own control in this longitudinal study, we also used univariate analysis in a targetted approach for individual microRNAs. The majority of circulating microRNAs remained unaltered following RYGB. However, 48 out of the 159 detected microRNAs were differentially expressed in at least one postoperative timepoint compared to preoperative levels (Fig. 1). At 1 month following surgery circulating microRNA levels were largely unchanged, with only 1 microRNA significantly increased and 1 microRNA significantly decreased relative to preoperative levels. At 3 months only 1 microRNA was significantly increased and 4 microRNAs were significantly decreased relative to preoperative levels. The number of differentiated microRNAs continued to increase with each sequential timepoint following RYGB, rising to 10 at 6 months (4 increased, 6 decreased). At 9 months the expression of 28 microRNAs were significantly altered (1 increased, 27 decreased) and at 12 months 31 microRNAs were significantly altered (1 increased, 30 decreased) compared to preoperative expression. A series of volcano plots of circulating microRNA expression at each postoperative timepoint relative to preoperative levels can be found in Fig. 2.

**Fig. 3:**
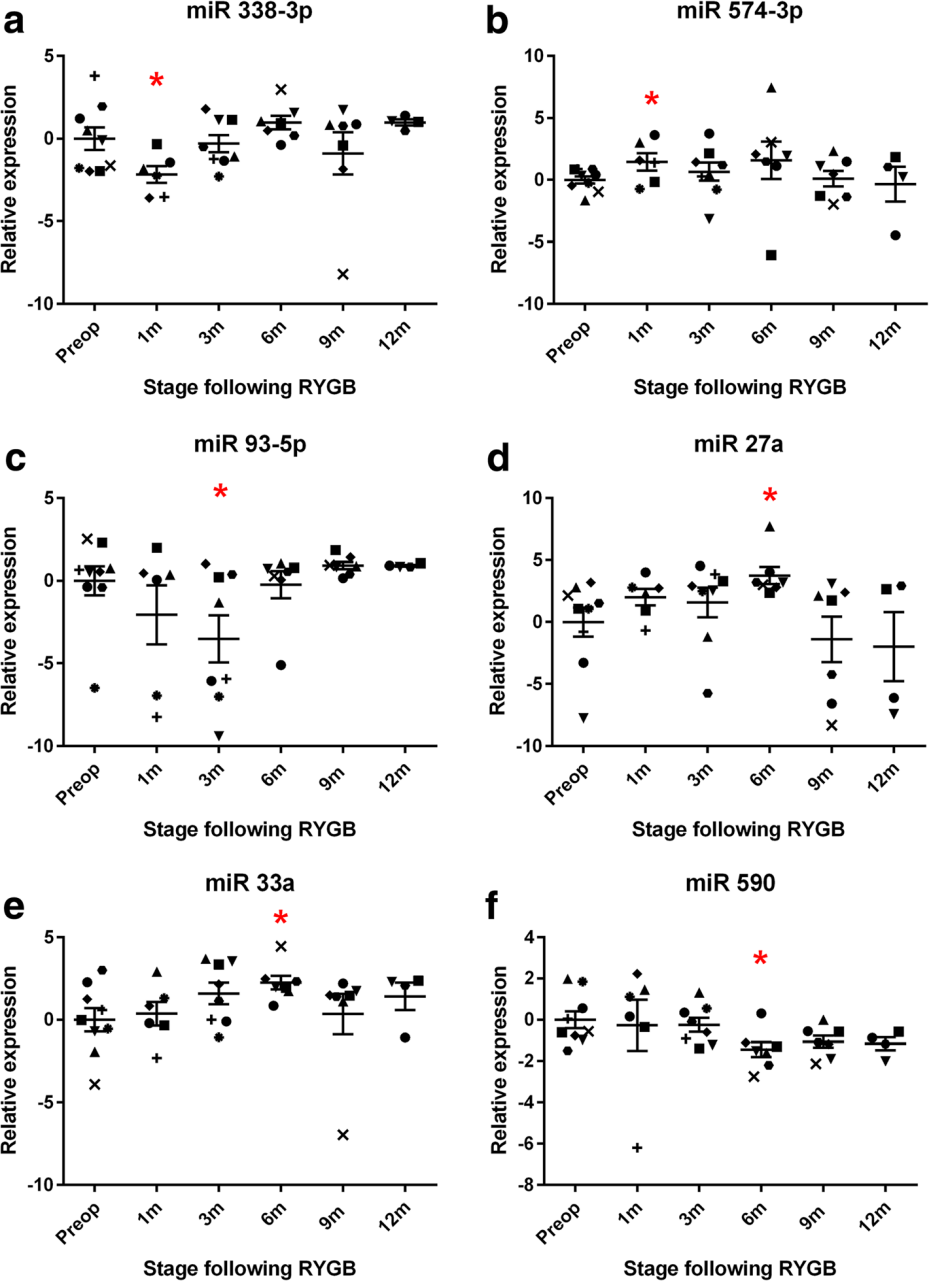
The early bariatric circulating microRNA response showing temporal selectivity. Normalised logarithmic relative preoperative and postoperative expression of differentiated microRNAs. **a** miR 338-3p, (**b**) miR 574-3p, (**c**) miR 93-5p, (**d**) miR 27a, (**e**) miR 33a, (**f**) miR 590. Data represents mean ± standard error of mean. *p < 0.05 by Student’s t test following Sidak-Bonferroni correction for multiple comparisons. Each patient is represented by a unique symbol

**Fig. 4:**
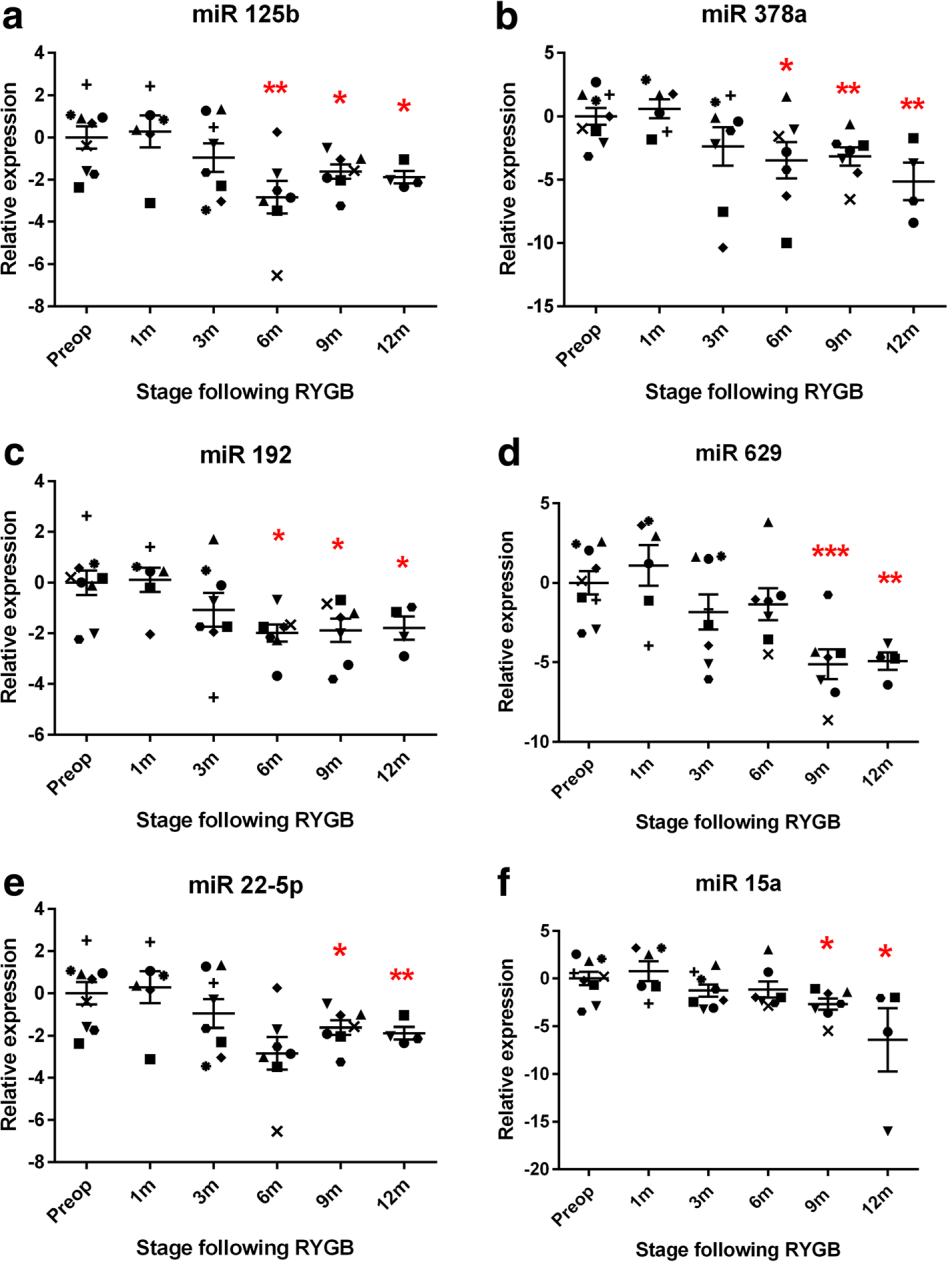
The late bariatric circulating microRNA response showing progressive temporal selectivity. Normalised logarithmic relative preoperative and postoperative expression of differentiated microRNAs. **a** miR 125b, (**b**) miR 378a, (**c**) miR 192, (**d**) miR 629, (**e**) miR 22-5p and (**f**) miR 15a. Data represents mean ± standard error of mean. *p < 0.05, **p < 0.01, ***p < 0.001 by Student’s t test following Sidak-Bonferroni correction for multiple comparisons. Each patient is represented by a unique symbol

These changes in circulating microRNA expression following RYGB were broadly characterized into 2 types of temporal response. The first early response was characterized by microRNAs whose expression was significantly different in the first months following RYGB before reverting to the preoperative baseline at the later months. MicroRNAs that followed this pattern included miR 338-3p, miR 93-5p and miR 590, whose levels significantly decreased in the early months following surgery, as well as miR 547-3p, miR 27a and mR 33a, whose levels increased (Fig. 3). However, most microRNAs differentially expressed following RYGB remained unchanged in the early months following surgery. Compared to preoperative levels of expression, their expression significantly altered 6 or 9 months following surgery and this was maintained and in some cases enhanced at 12 months. These microRNAs comprised the sustained late response. The most prominent microRNA that followed this pattern was miR 15a, whose expression at 3 months and 6 months remained comparable to preoperative levels but at 9 months miR 15a levels decreased significantly 6.52 fold and decreased further to 85 fold at 12 months. Similar patterns were found with circulating levels of miR 125b, miR 378a, miR 192, miR 629 and miR 22-5p, all were downregulated following RYGB (Fig. 4). All microRNAs whose expression was significantly different following RYGB are listed in Fig. 1 and all detected microRNA expression data is tabulated in Additional file 3: Table S1.

### Correlations with clinical outcomes

Circulating microRNAs levels correlated with clinical measurements (Additional file 4: Table S2). A total of 16 microRNAs significantly correlated with BMI. Expression levels of seven microRNAs were positively correlated with BMI including miR 148a-3p and miR 125b. In contrast circulating levels of nine microRNAs demonstrated a negative correlation with BMI including miR 33a. The expression of 19 circulating microRNAs significantly correlated with the percentage of excess preoperative weight lost. All but two (miR 301a and miR 374b) correlated negatively. Nine microRNAs significantly correlated with fasting blood glucose, including miR 320a and miR 590-5p. Only one microRNA (miR 346) correlated positively with fasting blood glucose levels. Additionally, 17 microRNAs significantly correlated with age.

### Pathway analysis

**Fig. 5:**
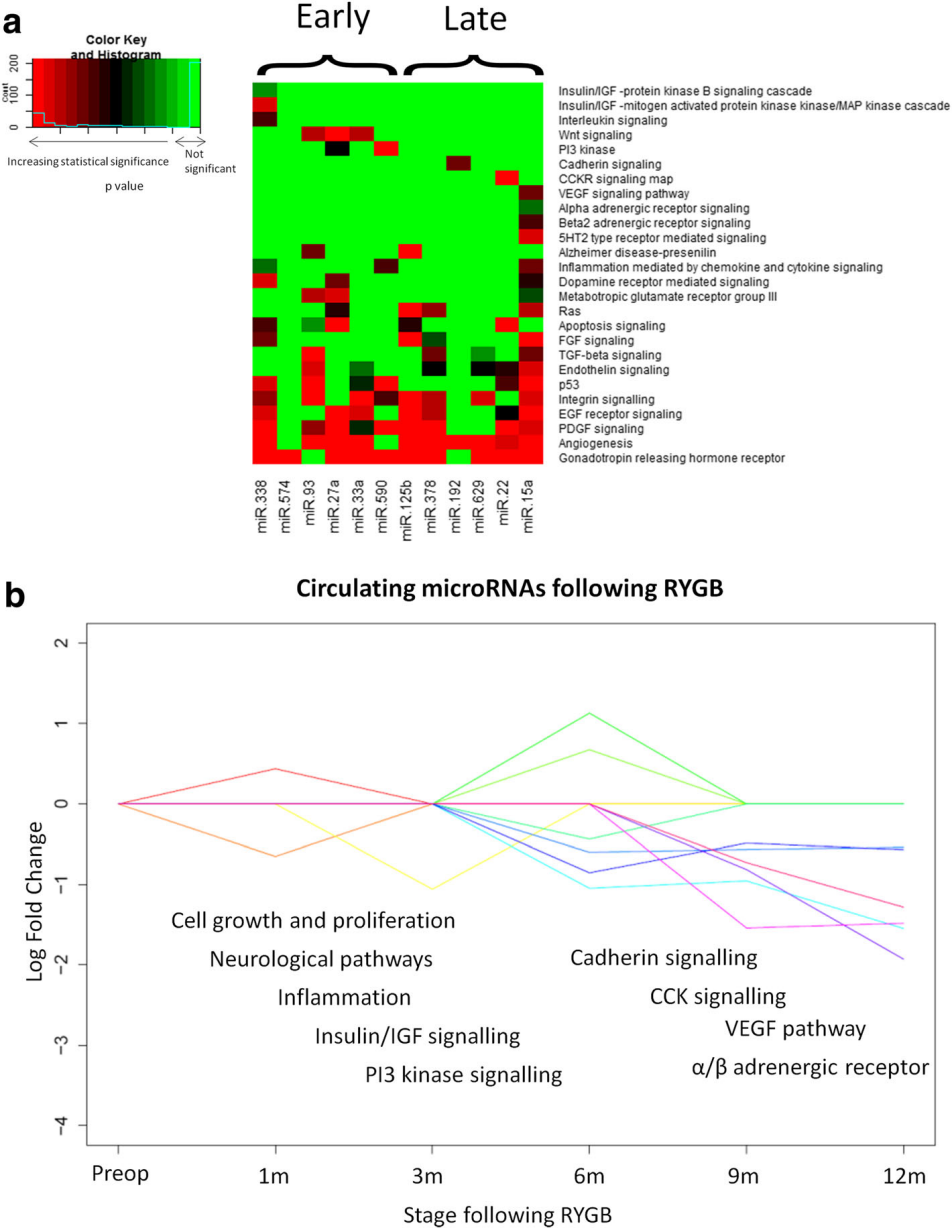
Pathway analysis. **a** Comparative heatmap of *p* values of pathways predicted to be regulated by microRNAs in the early and late response following RYGB. Increasing statistical significance is indicated by the shift in colour from green to red. **b** The circulating microRNA response following RYGB and some of the biological processes they’re predicted to regulate. Each line and shade of colour represents a circulating microRNA in the early and late microRNA responses following RYGB

To determine the potential biological consequences of changes in the expression of circulating microRNAs that made up the early and late post-bariatric responses, their mRNA targets and associated metabolic pathways were predicted using miRWalk and PANTHER bioinformatics (Fig. 5). The regulation of pathways involved in cell growth and proliferation, inflammation and neurological processes were predicted in both the early and late stages of response. The early response was also characterized by the predicted regulation of insulin/IGF pathways and the PI3 kinase pathway. Predicted pathways unique to the late stage of response were the cholecystokinin receptor pathway, α/β-adrenergic receptor pathway, cadherin signaling and the VEGF signaling pathway.

## Discussion

We have shown for the first time that RYGB persistently and progressively modulates circulating microRNA expression in a temporal manner with an increasing departure in profile from the preoperative obese state. In total, RYGB led to the differentiated expression of 48 circulating microRNAs typified by the selective, time-dependent differential expression of specific microRNAs. A temporary, immediate response is followed by a longer-lasting late response.

The circulating microRNAs whose expression is modified following RYGB are predicted to regulate cell growth, inflammation and neurological processes, and at the later months receptor pathways involved in gut hormone signaling, lipolysis and diabetic kidney disease. Although pathway analysis was largely predicted computationally, many of the circulating microRNAs whose expression is modified by RYGB have been experimentally linked with the metabolic syndrome. MiR 93, shown here to be decreased in the first few months following surgery, inhibits glucose transport and is overexpressed in insulin-resistant women with polycystic ovary syndrome [19]. MiR 27, another member of the early bariatric microRNA response, is a reported promoter of adipogenesis [20]. MiR 15a, decreased an astonishing 85 fold 1 year following surgery, also promotes adipogenesis [21] and miR 192 has a central regulatory role in diabetic nephropathy [22]. The altered expression of these microRNAs following RYGB may explain some of the mechanisms behind the health benefits associated with bariatric surgery.

The early postoperative months demonstrated the fewest changes in circulating microRNA expression, but the number increased longitudinally. Interestingly, as illustrated by the PCA scores plot of circulating microRNA profiles (Additional file 2: Figure S2), at 1 month following RYGB the microRNA circulating profile shifts from the preoperative profile, before a pronounced and gradual trajectory shift in the opposite direction from 3 months onwards. A possible explanation is that the initial physiological response to bariatric surgery was homeostatic as the body attempted to maintain the *status quo*. There is increasing evidence to suggest microRNAs help to maintain system robustness, fine-tuning or ‘buffering’ gene expression in response to subtle internal or external stimuli [23]. However, once a tipping point has been reached this homeostatic safety mechanism was adjusted and reset, moving the individual physiologically and phenotypically further from the morbidly obese state. In essence, this progressive timeline shift of circulating microRNA expression reveals a view of the physiological trajectory of bariatric surgery.

Many of the circulating microRNAs whose expression has been found to be altered by RYGB in the current study have also been reported as circulating biomarkers of T2DM, including of miR 15a, miR 192, miR 130b and miR 125b [24–26]. The loss of circulating miR 126 in particular is reportedly a strong selector for T2DM [25]. MiR 126 expression increased over 10 fold in each of the first 4 postoperative time points relative to preoperative levels, although none of the increases reached statistical significance (Additional file 3: Table S1). Importantly, fasting blood glucose levels positivity correlated with circulating miR 320a (Additional file 3: Table S2), consistent with previous findings [26] and supporting its candidacy as a novel diabetic biomarker. Circulating expression of miR 320a also correlated negatively with weight loss. In total, circulating levels of 9 microRNAs significantly correlated with fasting blood glucose levels. Correlations between circulating microRNAs levels and age were also found, supporting previous studies that show age impacts microRNA levels [27].

Although previous studies [9, 11, 12] have reported that bariatric surgery leads to changes in the expression of circulating microRNAs, these were time snapshots and lacked the temporal dependencies we have uncovered in the present longitudinal study. Lirun et al. looked at the effect of Roux-en-Y gastric bypass on circulating microRNAs 3 months postoperatively in diabetic patients, divided into a low BMI and high BMI preoperative groups [11]. They reported that 39 microRNAs were differentially expressed after surgery across both groups. Both *Lirun* et al. and our study reported the downregulation of let 7, miR 93 and miR 106b expression 3 months postoperatively, while the decrease in miR 16 expression at 3 months in *Lirun* et al. was only significant in our study after 9 months. Interestingly, they reported differences in the postoperative changes in microRNAs in the low and high BMI groups, suggesting that changes in microRNAs following surgery may depend on preoperative conditions.

Two further studies assessed circulating microRNA expression before and 12 months after RYGB. Ortega et al. [9] reported a significant modulation of 14 circulating microRNAs including decreases in miR 125b and miR 16 and an increase in miR 221, mirroring our findings (miR 211 increased across all postoperative timepoints, albeit not significantly). However, they also reported significant increases in the circulating levels of miR 130b and miR 21. The expression of both microRNAs were significantly decreased in our study 12 months following RYGB. Ortega et al. were also unable to detect a change in miR 15a following surgery. In another study, Hubal et al. reported the modified expression of 168 exosome-derived circulating microRNAs 1 year following RYGB and illustrated a correlation between altered microRNAs and improvements in insulin resistance [12]. Filtering for microRNAs with validated targets in insulin signaling or that correlated with changes insulin resistance, they identified several surgery-responsive microRNAs, including miR 125b and miR 122. However, it is important to reiterate that each of these previous studies measured circulating microRNAs at a single postoperative timepoint and are therefore merely snapshots of a dramatically altering physiology. Our longitudinal study emphasizes that the post-bariatric surgery circulating microRNAome and associated changes of physiology are both dynamic.

In rats, circulating levels of miR 122 decrease 60 fold following RYGB compared to sham-operated animals [15]. MiR 122 is a liver microRNA responsible for regulating lipid metabolism [28] and its decrease leads to increased glucose transportation, accelerated glycolysis and the inhibition of gluconeogenesis following RYGB [15]. This is consistent with the recent discovery that RYGB reprograms intestinal glucose metabolism by increasing glycolysis and glucose uptake [29], and suggests miR 122 could contribute to this effect. The present study also found a consistent decrease in circulating miR 122 in humans following RYGB from 3 months onwards, although this did not achieve statistical significance (Additional file 3: Table S1). Other microRNAs that decreased following RYGB in rats include miR 93, miR 30e and miR 320 [15] and we report significant decreases in the human orthologs of these microRNAs in our study.

The role of microRNAs following bariatric surgery remains relatively unexplored and offers potential insights into the dynamic physiological changes that follow surgical intervention. Our study, as with the few that have preceded it, was limited to a small number of participating individuals and should be considered a pilot study at this stage. Additionally, not all subjects provided samples at all post-operative timepoints. Nevertheless, our study confirms the proof of principle that analysis of circulating microRNAs offers insight into the dynamic physiological changes associated with bariatric intervention. Well-designed, multi-center studies with larger diabetes subsets, non-bariatric obese and lean control groups and more robust clinical follow-ups and endpoints would provide a clearer picture into the role microRNAs play following bariatric surgery. Although the field is still in its infancy, bariatric surgery has been consistently shown to significantly alter circulating microRNA levels. MicroRNA expression profiles offer a wealth of biological information and mechanistic insights, as the changes in the regulation of several core physiological processes are related to the expression of the microRNAs that regulate them. MicroRNAs can act as mediators or effectors in positive and negative feedback loops in wider regulation networks. Their presence in circulation in exosomes and high-density lipoprotein or argonaute complexes that can be taken up in an active form by recipient cells is also suggestive of a potential tissue-to-tissue communicative role and that microRNAs may have hormonal as well as biomarker potential [30].

## Conclusions

We have shown here that RYGB fundamentally alters microRNA expression in the circulation in a temporal manner with an increasing departure from the preoperative state. These microRNAs correlate with the beneficial physiological and metabolic fluxes seen after these operations and they therefore represent potentially novel post-surgical biomarkers of surgical outcomes. There is ongoing debate on updating the criteria for bariatric surgery, in recognition of its ability to improve health beyond weight loss. MicroRNA expression profiles could potentially add another facet for consideration in the decision-pathway for bariatric operations and offer additional opportunity for developing the next generation of combined multi-modal metabolic interventions to enhance the treatment outcomes for obesity and its associated disorders.

## Additional files

Additional file 1: **Figure S1.** Normalisation of profiling data. (A) Coefficient of variance against cumulative distribution for each expressed microRNA either not normalised (black), normalised to global mean (blue), miR 223-3p and miR 26a (red), miR 101-3p and miR 19a (purple) or a combination of miR 223-3p, miR 26a, miR 101-3p and miR 19a (green). (B) Mean coefficient of variance of the 50% least variable microRNAs for the four different normalisation methods. Data represent mean ± SEM. ***p < 0.001, ****p < 0.0001 by Student’s t-test. (C) The most stable microRNAs as determined by the geNorm and Normfinder algoritms. M-Value is the geNorm stability value, defined as the variation of a microRNA compared to all other microRNAs. SD is the Normfinder stability value, calculated as the sum of the estimated intragroup and intergroup variation. (D) Boxplots of raw Cq values of the 4 endogenous microRNAs used to normalise microRNA expression. (TIF 1470 kb)

Additional file 2: **Figure S2.** Multivariate analysis of profiling data. (A) Principal Component Analysis (PCA) scores plot of bariatric circulating microRNA preoperative (pink), 1 month postoperative (red), 3 month postoperative (green), 6 month postoperative (light blue), 9 month postoperative (blue) and 12 month postoperative (black) profiles. (B) Trajectory PCA scores plot of mean preoperative and postoperative components ± standard deviation. (C) Comparative heat map of preoperative and postoperative mean relative microRNA expression and (D) associated cluster dendrogram. (TIF 2531 kb)

Additional file 3: **Table S1.** Post-bariatric circulating microRNA fold changes relative to preoperative levels. (DOCX 48 kb)

Additional file 4: **Table S2.** Significant Pearson correlations between circulating microRNA expression and measured clinical parameters. (DOCX 20 kb)

## Abbreviations

BMI: Body mass index
CV: Coefficient of variances
LNA: Locked nucleic acid
mRNA: Messenger ribonucleic acid
NICE: National Institute of Health and Care Excellence
PCA: Principal component analysis
RYGB: Roux-en-Y Gastric Bypass
T2DM: Type 2 diabetes mellitus
