# Supplementary figures and images for: Improved physiology and metabolic flux after Roux-en-Y gastric bypass is associated with temporal changes in the circulating microRNAome: a longitudinal study in humans
Source: BMC Obes. 2018 May 31;5:20. doi: 10.1186/s40608-018-0199-z (PMC5984421; doi:10.1186/s40608-018-0199-z)

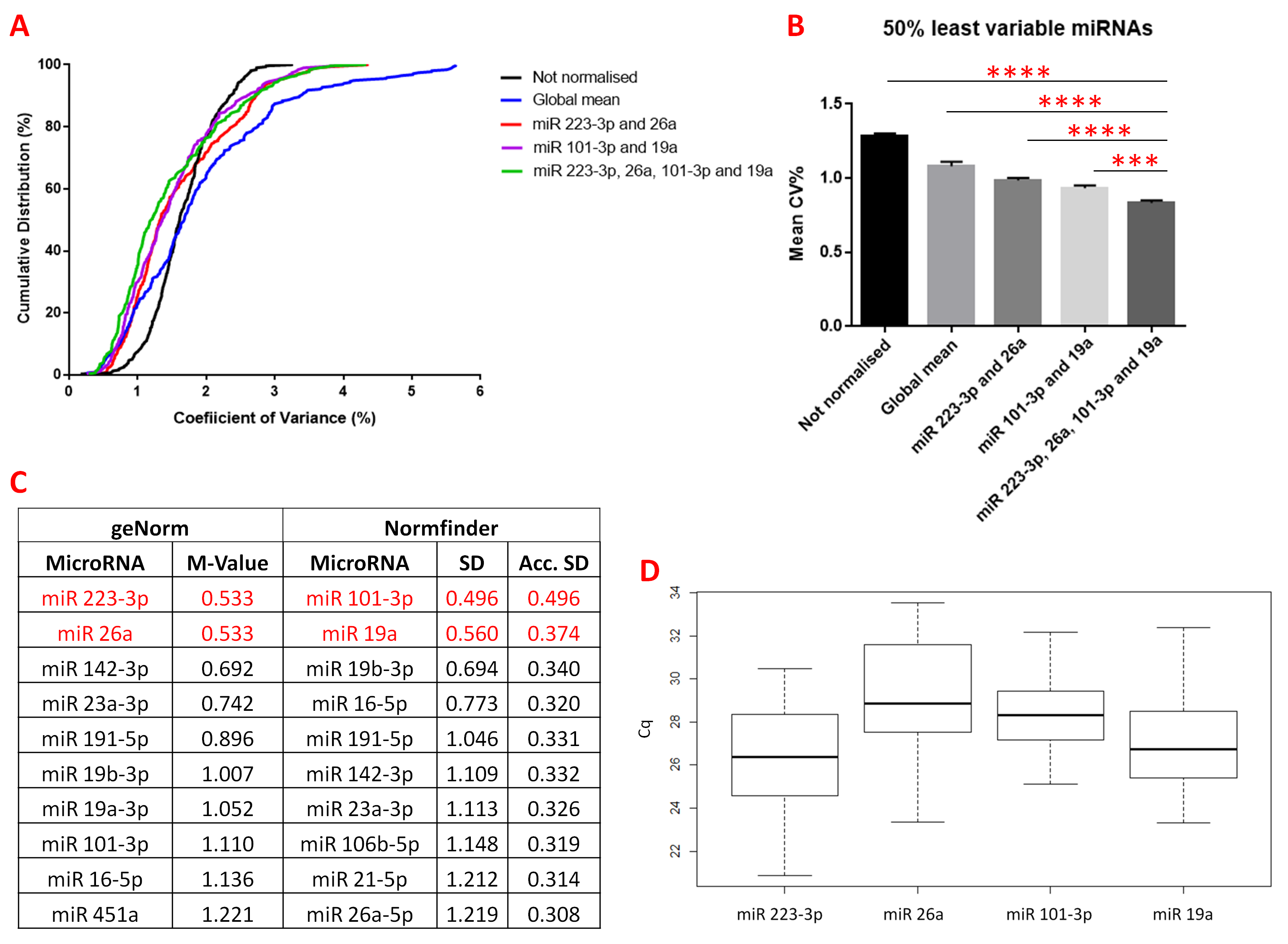

Supplement: Supplementary file 1 — Figure S1. Normalisation of profiling data. (A) Coefficient of variance against cumulative distribution for each expressed microRNA either not normalised (black), normalised to global mean (blue), miR 223-3p and miR 26a (red), miR 101-3p and miR 19a (purple) or a combination of miR 223-3p, miR 26a, miR 101-3p and miR 19a (green). (B) Mean coefficient of variance of the 50% least variable microRNAs for the four different normalisation methods. Data represent mean ± SEM. ***p < 0.001, ****p < 0.0001 by Student’s t-test. (C) The most stable microRNAs as determined by the geNorm and Normfinder algoritms. M-Value is the geNorm stability value, defined as the variation of a microRNA compared to all other microRNAs. SD is the Normfinder stability value, calculated as the sum of the estimated intragroup and intergroup variation. (D) Boxplots of raw Cq values of the 4 endogenous microRNAs used to normalise microRNA expression. (TIF 1470 kb) [file 40608_2018_199_MOESM1_ESM.tif]

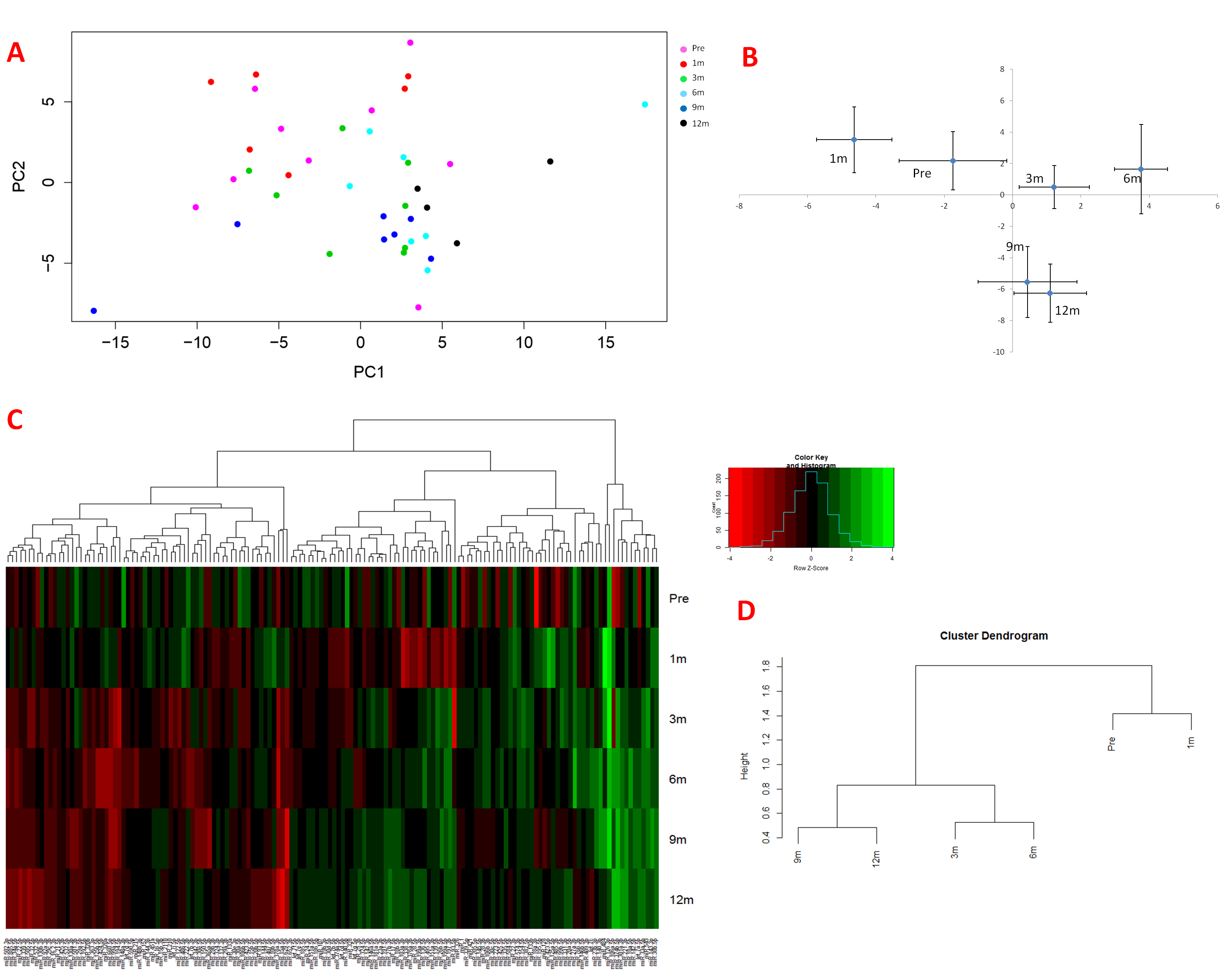

Supplement: Supplementary file 2 — Figure S2. Multivariate analysis of profiling data. (A) Principal Component Analysis (PCA) scores plot of bariatric circulating microRNA preoperative (pink), 1 month postoperative (red), 3 month postoperative (green), 6 month postoperative (light blue), 9 month postoperative (blue) and 12 month postoperative (black) profiles. (B) Trajectory PCA scores plot of mean preoperative and postoperative components ± standard deviation. (C) Comparative heat map of preoperative and postoperative mean relative microRNA expression and (D) associated cluster dendrogram. (TIF 2531 kb) [file 40608_2018_199_MOESM2_ESM.tif]
