# Supplementary material for: Improved physiology and metabolic flux after Roux-en-Y gastric bypass is associated with temporal changes in the circulating microRNAome: a longitudinal study in humans
Source: BMC Obes. 2018 May 31;5:20. doi: 10.1186/s40608-018-0199-z (PMC5984421; doi:10.1186/s40608-018-0199-z)
Supplement: Supplementary file 3 — Table S1. Post-bariatric circulating microRNA fold changes relative to preoperative levels. (DOCX 48 kb) [file 40608_2018_199_MOESM3_ESM.docx]

| **Table S1: Post-bariatric circulating microRNA fold changes relative to preoperative levels^ⱡ^** | | | | | | | | | | |
| --- | --- | --- | --- | --- | --- | --- | --- | --- | --- | --- |
| **MicroRNA** | **1 month** | | **3 months** | | **6 months** | | **9 months** | | **12 months** | |
|  | **FC** | **p** | **FC** | **p** | **FC** | **p** | **FC** | **p** | **FC** | **p** |
| **let-7a-5p** | -1.192 | 0.901 | 1.259 | 0.865 | 2.500 | 0.433 | 2.310 | 0.476 | 2.575 | 0.546 |
| **let-7b-3p** | 1.142 | 0.886 | 1.111 | 0.905 | -3.172 | 0.145 | -5.349 | 0.081 | -1.505 | 0.629 |
| **let-7b-5p** | -2.229 | 0.269 | **-1.827** | **0.044** | -1.335 | 0.291 | 1.057 | 0.886 | -1.213 | 0.585 |
| **let-7c** | 1.083 | 0.942 | 1.718 | 0.521 | 4.297 | 0.133 | -1.002 | 0.990 | -5.706 | 0.160 |
| **let-7d-3p** | 1.299 | 0.557 | -1.729 | 0.286 | -2.003 | 0.172 | **-2.147** | **0.038** | **-2.760** | **0.035** |
| **let-7d-5p** | -2.045 | 0.328 | -1.714 | 0.336 | 1.683 | 0.405 | 1.079 | 0.875 | -1.240 | 0.750 |
| **let-7e-5p** | -1.123 | 0.884 | -1.169 | 0.774 | -1.301 | 0.629 | -1.983 | 0.341 | 1.039 | 0.959 |
| **let-7f-5p** | -1.071 | 0.893 | -1.303 | 0.664 | -1.509 | 0.581 | -1.214 | 0.760 | -1.481 | 0.695 |
| **let-7g-5p** | 1.065 | 0.782 | -1.138 | 0.551 | -2.554 | 0.255 | 1.176 | 0.429 | -1.081 | 0.745 |
| **let-7i-5p** | 1.903 | 0.102 | -2.051 | 0.087 | **-1.921** | **0.039** | **-2.154** | **0.032** | -2.054 | 0.091 |
| **miR-1** | -1.225 | 0.663 | 1.354 | 0.594 | 1.369 | 0.648 | -2.567 | 0.234 | -2.600 | 0.170 |
| **miR-103a-3p** | -5.757 | 0.085 | -1.123 | 0.693 | 1.294 | 0.379 | 1.051 | 0.877 | 1.314 | 0.479 |
| **miR-106a-5p** | 3.763 | 0.459 | -3.164 | 0.558 | 9.581 | 0.142 | 1.392 | 0.861 | 11.202 | 0.242 |
| **miR-106b-3p** | 1.137 | 0.890 | 2.289 | 0.301 | 2.411 | 0.415 | -3.096 | 0.205 | -1.533 | 0.661 |
| **miR-106b-5p** | -1.031 | 0.956 | **-2.247** | **0.028** | -1.002 | 0.990 | -2.425 | 0.103 | **-3.740** | **0.040** |
| **miR-107** | -1.031 | 0.951 | -1.184 | 0.674 | -1.082 | 0.863 | -1.004 | 0.990 | 1.132 | 0.797 |
| **miR-10b-5p** | 4.244 | 0.193 | -1.299 | 0.742 | -1.376 | 0.708 | -1.261 | 0.798 | -9.328 | 0.078 |
| **miR-122-5p** | 1.911 | 0.343 | -3.666 | 0.092 | -1.382 | 0.572 | -2.554 | 0.195 | -4.186 | 0.070 |
| **miR-125a-5p** | 6.584 | 0.059 | 1.009 | 0.988 | 1.216 | 0.715 | 1.191 | 0.770 | 1.190 | 0.771 |
| **miR-125b-5p** | 1.221 | 0.755 | -1.943 | 0.280 | **-7.144** | **0.008** | **-3.067** | **0.033** | **-3.701** | **0.048** |
| **miR-126-3p** | 12.599 | 0.167 | 13.881 | 0.099 | 12.412 | 0.136 | 12.175 | 0.139 | 1.355 | 0.907 |
| **miR-128** | 3.660 | 0.288 | -1.741 | 0.642 | 1.092 | 0.938 | -4.261 | 0.278 | -1.767 | 0.695 |
| **miR-130a-3p** | 2.410 | 0.470 | -1.297 | 0.850 | 1.300 | 0.819 | -8.377 | 0.122 | -6.195 | 0.280 |
| **miR-130b-3p** | -1.115 | 0.875 | -2.095 | 0.267 | -1.082 | 0.904 | -2.268 | 0.240 | **-13.871** | **0.006** |
| **miR-132-3p** | -1.593 | 0.654 | -1.247 | 0.834 | -1.003 | 0.990 | -1.468 | 0.698 | -1.966 | 0.544 |
| **miR-133a** | -1.762 | 0.362 | -1.810 | 0.489 | 1.613 | 0.599 | -3.487 | 0.148 | -3.521 | 0.175 |
| **miR-133b** | 1.412 | 0.743 | 1.826 | 0.568 | 3.551 | 0.256 | 1.127 | 0.918 | -1.429 | 0.789 |
| **miR-136-5p** | -3.043 | 0.198 | 1.447 | 0.603 | 3.975 | 0.118 | -1.483 | 0.702 | 1.305 | 0.771 |
| **miR-139-5p** | -2.385 | 0.291 | 2.421 | 0.205 | 2.858 | 0.147 | 1.008 | 0.990 | 1.250 | 0.787 |
| **miR-140-3p** | 2.075 | 0.665 | 1.264 | 0.878 | 6.278 | 0.201 | 3.633 | 0.340 | -1.263 | 0.903 |
| **miR-140-5p** | -2.505 | 0.200 | -2.639 | 0.171 | 1.580 | 0.468 | -1.877 | 0.437 | -1.852 | 0.415 |
| **miR-142-3p** | -1.237 | 0.565 | 1.216 | 0.557 | 1.267 | 0.470 | -1.102 | 0.781 | 1.419 | 0.412 |
| **miR-142-5p** | 1.113 | 0.926 | 1.241 | 0.824 | 3.164 | 0.363 | 2.086 | 0.500 | 1.345 | 0.828 |
| **miR-143-3p** | 3.094 | 0.252 | 1.422 | 0.699 | 2.561 | 0.271 | 3.574 | 0.159 | 1.913 | 0.602 |
| **miR-144-3p** | 2.228 | 0.200 | -1.295 | 0.631 | -1.232 | 0.678 | -1.705 | 0.445 | -2.002 | 0.413 |
| **miR-144-5p** | 2.636 | 0.306 | -1.208 | 0.816 | 1.490 | 0.693 | -2.913 | 0.287 | -7.657 | 0.090 |
| **miR-145-5p** | -1.047 | 0.957 | 1.262 | 0.653 | 1.751 | 0.339 | -2.753 | 0.295 | -1.319 | 0.786 |
| **miR-146a-5p** | -1.208 | 0.725 | 1.660 | 0.227 | 1.302 | 0.546 | -1.112 | 0.813 | 1.249 | 0.681 |
| **MicroRNA** | **1 month** | | **3 months** | | **6 months** | | **9 months** | | **12 months** | |
|  | **FC** | **p** | **FC** | **p** | **FC** | **p** | **FC** | **p** | **FC** | **p** |
| **miR-146b-5p** | -1.156 | 0.804 | 1.596 | 0.256 | 2.829 | 0.147 | -2.082 | 0.273 | -2.454 | 0.179 |
| **miR-148a-3p** | 1.278 | 0.648 | -1.717 | 0.230 | -2.309 | 0.076 | **-2.575** | **0.038** | -3.092 | 0.061 |
| **miR-148b-3p** | 1.349 | 0.420 | 1.047 | 0.880 | -1.501 | 0.151 | **-1.862** | **0.050** | -1.967 | 0.077 |
| **miR-150-5p** | 1.129 | 0.871 | 1.506 | 0.576 | -1.249 | 0.788 | -9.519 | 0.099 | -2.036 | 0.424 |
| **miR-151a-3p** | -2.391 | 0.157 | 1.563 | 0.364 | 2.160 | 0.230 | -1.100 | 0.859 | -1.147 | 0.849 |
| **miR-151a-5p** | -1.117 | 0.683 | -1.630 | 0.496 | 1.099 | 0.642 | 1.109 | 0.681 | 1.191 | 0.523 |
| **miR-152** | 1.451 | 0.541 | -1.061 | 0.908 | -1.637 | 0.248 | -1.410 | 0.372 | -2.125 | 0.140 |
| **miR-154-5p** | 1.192 | 0.839 | 3.330 | 0.095 | 2.868 | 0.291 | -1.031 | 0.969 | -2.193 | 0.451 |
| **miR-15a-5p** | 1.710 | 0.528 | -2.412 | 0.200 | -2.226 | 0.301 | **-6.522** | **0.013** | **-84.905** | **0.019** |
| **miR-15b-3p** | 1.628 | 0.712 | -2.457 | 0.448 | -1.092 | 0.939 | -1.183 | 0.876 | -2.801 | 0.468 |
| **miR-15b-5p** | 1.220 | 0.766 | -1.162 | 0.769 | 1.412 | 0.612 | -2.087 | 0.309 | -8.795 | 0.062 |
| **miR-16-2-3p** | -1.074 | 0.939 | -1.642 | 0.412 | -1.897 | 0.465 | **-4.056** | **0.045** | -5.841 | 0.078 |
| **miR-16-5p** | 1.370 | 0.493 | -1.696 | 0.183 | -1.415 | 0.380 | -1.117 | 0.799 | -1.375 | 0.542 |
| **miR-17-5p** | -1.524 | 0.545 | -1.437 | 0.480 | 1.881 | 0.279 | 1.297 | 0.594 | 1.411 | 0.600 |
| **miR-181a-5p** | -1.039 | 0.954 | 1.249 | 0.700 | 1.034 | 0.950 | 1.213 | 0.680 | 1.147 | 0.825 |
| **miR-185-5p** | -1.054 | 0.931 | -1.528 | 0.112 | -1.058 | 0.835 | -1.507 | 0.314 | -1.098 | 0.764 |
| **miR-186-5p** | -1.156 | 0.830 | -1.199 | 0.751 | 1.353 | 0.561 | -1.428 | 0.654 | 1.203 | 0.779 |
| **miR-18a-3p** | 2.397 | 0.168 | -1.104 | 0.810 | 1.179 | 0.834 | -4.555 | 0.056 | -1.253 | 0.670 |
| **miR-18a-5p** | 1.114 | 0.925 | 1.483 | 0.692 | 6.756 | 0.078 | 1.396 | 0.785 | 1.706 | 0.682 |
| **miR-18b-5p** | -1.326 | 0.710 | -1.237 | 0.737 | 1.743 | 0.255 | 2.002 | 0.160 | 1.868 | 0.333 |
| **miR-191-5p** | -1.136 | 0.806 | 1.577 | 0.194 | 1.504 | 0.256 | 1.351 | 0.415 | 1.301 | 0.574 |
| **miR-192-5p** | 1.084 | 0.873 | -2.103 | 0.206 | **-3.976** | **0.007** | **-3.680** | **0.016** | **-3.458** | **0.047** |
| **miR-194-5p** | -1.935 | 0.297 | **-6.792** | **0.001** | -3.465 | 0.141 | **-7.387** | **0.006** | **-8.758** | **0.003** |
| **miR-195-5p** | 2.764 | 0.255 | -1.308 | 0.709 | -1.176 | 0.787 | -3.095 | 0.124 | -1.107 | 0.883 |
| **miR-197-3p** | -2.506 | 0.350 | 3.074 | 0.181 | 4.635 | 0.084 | 2.776 | 0.267 | 1.503 | 0.733 |
| **miR-199a-3p** | -4.064 | 0.210 | 1.532 | 0.475 | 2.062 | 0.248 | -1.469 | 0.673 | 1.511 | 0.619 |
| **miR-199a-5p** | -4.574 | 0.144 | 1.717 | 0.511 | 5.884 | 0.065 | 1.493 | 0.653 | 1.348 | 0.782 |
| **miR-19b-3p** | 1.049 | 0.890 | -1.061 | 0.821 | -1.081 | 0.778 | 1.092 | 0.780 | -1.015 | 0.967 |
| **miR-205-5p** | 1.458 | 0.689 | 1.011 | 0.990 | 1.981 | 0.558 | -2.540 | 0.352 | -2.127 | 0.526 |
| **miR-20a-5p** | 1.847 | 0.498 | 1.429 | 0.638 | 1.521 | 0.614 | -1.958 | 0.666 | 2.068 | 0.509 |
| **miR-20b-5p** | 1.502 | 0.581 | -1.009 | 0.989 | 1.626 | 0.565 | -1.564 | 0.541 | **-5.580** | **0.050** |
| **miR-210** | 1.695 | 0.519 | -3.081 | 0.123 | -1.268 | 0.811 | -2.526 | 0.343 | -2.331 | 0.375 |
| **miR-2110** | 1.107 | 0.900 | -1.383 | 0.607 | 1.151 | 0.858 | -1.296 | 0.685 | -3.563 | 0.146 |
| **miR-215** | 1.311 | 0.750 | -1.591 | 0.570 | -3.044 | 0.177 | -1.961 | 0.436 | -1.878 | 0.521 |
| **miR-21-5p** | 1.102 | 0.870 | -1.507 | 0.371 | -1.304 | 0.671 | **-2.993** | **0.026** | **-4.990** | **0.016** |
| **miR-221-3p** | 2.535 | 0.404 | 2.104 | 0.529 | 3.212 | 0.345 | 5.139 | 0.109 | 6.730 | 0.159 |
| **miR-222-3p** | 2.599 | 0.069 | -2.909 | 0.377 | -3.298 | 0.236 | -1.213 | 0.648 | 1.015 | 0.978 |
| **miR-223-5p** | 1.365 | 0.635 | -1.352 | 0.665 | 1.669 | 0.554 | -1.397 | 0.702 | -1.900 | 0.484 |
| **miR-22-3p** | 3.746 | 0.234 | 1.355 | 0.737 | -1.040 | 0.967 | -1.402 | 0.723 | -2.079 | 0.571 |
| **MicroRNA** | **1 month** | | **3 months** | | **6 months** | | **9 months** | | **12 months** | |
|  | **FC** | **p** | **FC** | **p** | **FC** | **p** | **FC** | **p** | **FC** | **p** |
| **miR-22-5p** | 1.437 | 0.609 | -1.558 | 0.396 | -1.958 | 0.305 | **-5.348** | **0.018** | **-19.128** | **0.002** |
| **miR-23a-3p** | 1.023 | 0.957 | 1.408 | 0.247 | 1.233 | 0.495 | 1.080 | 0.814 | 1.031 | 0.938 |
| **miR-23b-3p** | 1.449 | 0.721 | -1.637 | 0.697 | 5.006 | 0.120 | 2.213 | 0.403 | -2.331 | 0.568 |
| **miR-24-3p** | -1.188 | 0.613 | -1.952 | 0.505 | 1.022 | 0.936 | -1.111 | 0.719 | -1.099 | 0.791 |
| **miR-25-3p** | 1.022 | 0.986 | 1.193 | 0.838 | -2.120 | 0.538 | 1.184 | 0.861 | 1.266 | 0.848 |
| **miR-26b-5p** | -1.967 | 0.530 | 2.161 | 0.280 | 1.719 | 0.472 | 3.296 | 0.106 | -2.085 | 0.585 |
| **miR-27a-3p** | 4.039 | 0.216 | 2.994 | 0.363 | **13.533** | **0.023** | -2.634 | 0.513 | -3.982 | 0.443 |
| **miR-27b-3p** | -1.053 | 0.954 | -2.062 | 0.545 | -1.001 | 0.990 | 2.052 | 0.239 | 2.366 | 0.283 |
| **miR-28-3p** | -1.583 | 0.480 | 1.736 | 0.163 | 2.881 | 0.078 | **-3.387** | **0.027** | -1.631 | 0.507 |
| **miR-28-5p** | -2.393 | 0.365 | 1.025 | 0.974 | 2.220 | 0.417 | -2.022 | 0.476 | 1.507 | 0.708 |
| **miR-29a-3p** | -1.231 | 0.792 | -1.394 | 0.598 | 1.094 | 0.889 | -1.351 | 0.615 | -2.461 | 0.278 |
| **miR-29a-5p** | -1.474 | 0.502 | 1.175 | 0.784 | -1.119 | 0.818 | -3.828 | 0.066 | **-5.746** | **0.010** |
| **miR-29b-3p** | -1.836 | 0.317 | -1.462 | 0.410 | 1.202 | 0.751 | -1.866 | 0.279 | **-3.096** | **0.035** |
| **miR-29c-3p** | -1.012 | 0.983 | -1.888 | 0.142 | -1.436 | 0.562 | **-4.366** | **0.011** | **-7.437** | **0.005** |
| **miR-301a-3p** | -1.050 | 0.953 | 1.937 | 0.338 | **5.379** | **0.018** | 2.765 | 0.137 | **6.227** | **0.046** |
| **miR-30a-5p** | -1.926 | 0.413 | -2.347 | 0.318 | -1.221 | 0.743 | -1.963 | 0.439 | -3.178 | 0.123 |
| **miR-30b-5p** | 3.854 | 0.275 | 2.006 | 0.536 | 3.569 | 0.263 | 2.830 | 0.359 | 3.660 | 0.395 |
| **miR-30c-5p** | 1.195 | 0.692 | -1.211 | 0.695 | 1.290 | 0.502 | -3.454 | 0.219 | 1.217 | 0.632 |
| **miR-30d-5p** | -2.164 | 0.132 | -1.767 | 0.196 | -1.235 | 0.331 | -1.516 | 0.124 | -1.203 | 0.461 |
| **miR-30e-3p** | -2.089 | 0.132 | 1.082 | 0.851 | 1.837 | 0.257 | -2.612 | 0.103 | **-4.250** | **0.021** |
| **miR-30e-5p** | -1.716 | 0.478 | -1.605 | 0.300 | -1.967 | 0.351 | -7.573 | 0.100 | -1.033 | 0.829 |
| **miR-320a** | 2.079 | 0.313 | -1.752 | 0.383 | **-4.931** | **0.037** | **-5.058** | **0.007** | **-5.639** | **0.024** |
| **miR-320b** | 1.202 | 0.889 | -2.035 | 0.291 | -2.760 | 0.128 | **-3.363** | **0.040** | -4.339 | 0.059 |
| **miR-324-3p** | -1.861 | 0.425 | -1.577 | 0.405 | 1.393 | 0.712 | -1.303 | 0.703 | -4.435 | 0.105 |
| **miR-324-5p** | -1.686 | 0.354 | -1.049 | 0.905 | -1.445 | 0.504 | 1.272 | 0.564 | 1.082 | 0.888 |
| **miR-32-5p** | 1.634 | 0.523 | -3.121 | 0.091 | -1.499 | 0.569 | **-5.872** | **0.025** | **-11.797** | **0.008** |
| **miR-326** | -1.449 | 0.564 | 1.195 | 0.715 | 1.250 | 0.782 | -3.281 | 0.126 | -3.131 | 0.172 |
| **miR-328** | -1.210 | 0.800 | -2.304 | 0.351 | 2.169 | 0.318 | -1.635 | 0.406 | -1.384 | 0.674 |
| **miR-331-3p** | 2.114 | 0.302 | 2.159 | 0.192 | -1.009 | 0.990 | -2.479 | 0.286 | 2.114 | 0.384 |
| **miR-335-5p** | 1.859 | 0.440 | 1.239 | 0.771 | 1.004 | 0.990 | 1.379 | 0.563 | -1.983 | 0.382 |
| **miR-338-3p** | **-4.482** | **0.039** | -1.230 | 0.737 | 1.967 | 0.274 | -1.857 | 0.525 | 1.977 | 0.371 |
| **miR-339-3p** | -1.080 | 0.936 | -1.724 | 0.387 | -1.476 | 0.576 | -4.306 | 0.084 | **-11.956** | **0.016** |
| **miR-339-5p** | 1.020 | 0.979 | 1.420 | 0.550 | 1.249 | 0.743 | -2.095 | 0.331 | -2.041 | 0.422 |
| **miR-33a-5p** | 1.288 | 0.733 | 2.995 | 0.124 | **4.739** | **0.024** | 1.271 | 0.800 | 2.661 | 0.265 |
| **miR-342-3p** | -1.139 | 0.848 | 1.494 | 0.502 | -2.725 | 0.336 | -6.749 | 0.107 | -1.141 | 0.817 |
| **miR-34a-5p** | -1.200 | 0.875 | -2.275 | 0.426 | -3.108 | 0.284 | -5.222 | 0.167 | -13.774 | 0.087 |
| **miR-363-3p** | 1.176 | 0.830 | -2.919 | 0.055 | -2.838 | 0.265 | **-4.165** | **0.039** | **-6.015** | **0.037** |
| **miR-365a-3p** | -1.544 | 0.442 | -2.269 | 0.164 | -1.480 | 0.628 | **-14.393** | **0.000** | -2.425 | 0.224 |
| **miR-374a-5p** | -2.219 | 0.137 | -1.404 | 0.308 | 1.292 | 0.653 | -1.543 | 0.247 | -1.703 | 0.244 |
| **MicroRNA** | **1 month** | | **3 months** | | **6 months** | | **9 months** | | **12 months** | |
|  | **FC** | **p** | **FC** | **p** | **FC** | **p** | **FC** | **p** | **FC** | **p** |
| **miR-374b-5p** | 1.070 | 0.946 | **4.933** | **0.050** | **6.262** | **0.035** | **6.420** | **0.027** | 8.699 | 0.052 |
| **miR-376a-3p** | -3.139 | 0.184 | -1.012 | 0.987 | 1.954 | 0.412 | -1.624 | 0.563 | -1.247 | 0.821 |
| **miR-378a-3p** | 1.507 | 0.571 | -5.183 | 0.156 | **-11.137** | **0.032** | **-9.024** | **0.006** | **-35.243** | **0.003** |
| **miR-382-5p** | 1.166 | 0.869 | 1.627 | 0.571 | 1.897 | 0.565 | 1.144 | 0.895 | -1.696 | 0.675 |
| **miR-409-3p** | -2.077 | 0.332 | 1.068 | 0.907 | 2.599 | 0.176 | 1.217 | 0.797 | -2.966 | 0.270 |
| **miR-421** | 1.010 | 0.984 | 1.427 | 0.567 | 1.418 | 0.564 | -1.401 | 0.543 | -2.078 | 0.328 |
| **miR-423-3p** | -1.671 | 0.465 | -2.111 | 0.145 | -1.660 | 0.418 | **-4.642** | **0.021** | **-4.013** | **0.017** |
| **miR-423-5p** | 8.054 | 0.132 | 1.595 | 0.674 | 1.029 | 0.981 | -3.294 | 0.391 | -1.689 | 0.732 |
| **miR-424-5p** | 1.516 | 0.473 | -2.320 | 0.085 | -5.042 | 0.063 | **-3.069** | **0.029** | -2.873 | 0.099 |
| **miR-425-3p** | 1.609 | 0.472 | -1.355 | 0.481 | -1.085 | 0.901 | -2.939 | 0.086 | -3.326 | 0.155 |
| **miR-425-5p** | 1.547 | 0.718 | 1.154 | 0.886 | 2.494 | 0.436 | -1.470 | 0.751 | -1.240 | 0.878 |
| **miR-451a** | 1.716 | 0.448 | -2.016 | 0.282 | -1.727 | 0.374 | -1.271 | 0.731 | -1.558 | 0.592 |
| **miR-484** | 14.798 | 0.151 | 4.038 | 0.377 | 2.231 | 0.635 | 4.137 | 0.351 | 1.057 | 0.979 |
| **miR-485-3p** | 1.858 | 0.477 | 1.556 | 0.580 | 2.032 | 0.299 | -3.134 | 0.180 | -1.091 | 0.918 |
| **miR-486-5p** | 3.296 | 0.148 | -1.854 | 0.343 | -2.311 | 0.188 | -2.796 | 0.111 | -3.505 | 0.116 |
| **miR-495-3p** | -1.512 | 0.609 | 2.221 | 0.287 | 2.078 | 0.338 | -1.284 | 0.745 | -1.486 | 0.637 |
| **miR-497-5p** | 2.616 | 0.244 | 1.405 | 0.641 | 1.244 | 0.806 | -2.613 | 0.238 | **-7.824** | **0.039** |
| **miR-501-3p** | 1.183 | 0.877 | 1.693 | 0.569 | -1.393 | 0.742 | -6.811 | 0.057 | -7.161 | 0.115 |
| **miR-502-3p** | 1.335 | 0.681 | -2.393 | 0.135 | -3.069 | 0.129 | **-5.755** | **0.038** | **-13.228** | **0.004** |
| **miR-505-3p** | -1.029 | 0.981 | -3.760 | 0.265 | -1.625 | 0.660 | -5.377 | 0.190 | **-21.453** | **0.042** |
| **miR-532-3p** | -1.511 | 0.470 | -1.769 | 0.397 | 1.078 | 0.923 | -3.319 | 0.081 | **-16.700** | **0.007** |
| **miR-532-5p** | 1.171 | 0.828 | -2.052 | 0.167 | -1.199 | 0.848 | **-5.651** | **0.011** | -2.192 | 0.347 |
| **miR-543** | 1.909 | 0.485 | 4.442 | 0.117 | 5.423 | 0.091 | 2.410 | 0.267 | 2.268 | 0.376 |
| **miR-574-3p** | **2.743** | **0.049** | 1.581 | 0.393 | 2.996 | 0.264 | 1.076 | 0.872 | -1.267 | 0.740 |
| **miR-584-5p** | 2.759 | 0.303 | 2.068 | 0.443 | 1.450 | 0.687 | -1.451 | 0.670 | -3.960 | 0.244 |
| **miR-590-5p** | -1.202 | 0.816 | -1.190 | 0.648 | **-2.729** | **0.022** | -2.087 | 0.065 | -2.233 | 0.106 |
| **miR-629-5p** | 2.131 | 0.435 | -3.544 | 0.180 | -2.531 | 0.287 | **-34.772** | **0.001** | **-30.336** | **0.001** |
| **miR-652-3p** | -1.090 | 0.888 | -1.591 | 0.291 | -1.903 | 0.376 | -1.246 | 0.532 | -1.270 | 0.579 |
| **miR-660-5p** | 1.478 | 0.642 | -3.184 | 0.121 | -4.952 | 0.110 | **-12.738** | **0.003** | **-16.147** | **0.009** |
| **miR-766-3p** | -1.568 | 0.494 | 1.605 | 0.285 | 2.505 | 0.145 | -1.327 | 0.622 | **-3.765** | **0.044** |
| **miR-885-5p** | 1.413 | 0.737 | -2.183 | 0.474 | -2.155 | 0.512 | -6.271 | 0.076 | -15.316 | 0.050 |
| **miR-92a-3p** | 1.236 | 0.747 | -2.280 | 0.116 | -1.882 | 0.350 | **-4.509** | **0.015** | **-7.404** | **0.016** |
| **miR-93-3p** | 1.655 | 0.371 | 1.283 | 0.430 | 2.139 | 0.282 | -2.081 | 0.205 | **-5.518** | **0.007** |
| **miR-93-5p** | -4.168 | 0.272 | **-11.421** | **0.048** | -1.182 | 0.848 | 1.891 | 0.383 | 1.875 | 0.516 |
| **miR-99a-5p** | 1.772 | 0.461 | -5.110 | 0.064 | -4.344 | 0.055 | **-11.744** | **0.018** | **-6.782** | **0.047** |
| **miR-99b-5p** | 2.574 | 0.350 | 2.583 | 0.276 | 1.084 | 0.941 | -2.574 | 0.385 | 1.026 | 0.982 |

^ⱡ^Statistical significance was determined by Student’s t-test with Sidak-Bonferroni correction for multiple comparisons. Red denotes statistical significance.
