# Supplementary material for: Improved physiology and metabolic flux after Roux-en-Y gastric bypass is associated with temporal changes in the circulating microRNAome: a longitudinal study in humans
Source: BMC Obes. 2018 May 31;5:20. doi: 10.1186/s40608-018-0199-z (PMC5984421; doi:10.1186/s40608-018-0199-z)
Supplement: Supplementary file 4 — Table S2. Significant Pearson correlations between circulating microRNA expression and measured clinical parameters. (DOCX 20 kb) [file 40608_2018_199_MOESM4_ESM.docx]

Table S2: Significant Pearson correlations between circulating microRNA expression and measured clinical parameters^ⱡ^

| **BMI** | | | |
| --- | --- | --- | --- |
| **MicroRNA** | **r** | **p** | **Significance** |
| **miR-148a-3p** | 0.5195 | 0.0006 | *** |
| **miR-33a-5p** | -0.4107 | 0.0085 | ** |
| **miR-148b-3p** | 0.4008 | 0.0104 | * |
| **miR-136-5p** | -0.3881 | 0.0133 | * |
| **miR-191-5p** | -0.3831 | 0.0147 | * |
| **miR-199a-5p** | -0.3812 | 0.0152 | * |
| **let-7d-3p** | 0.3466 | 0.0285 | * |
| **miR-125b-5p** | 0.3457 | 0.0289 | * |
| **miR-107** | -0.3347 | 0.0348 | * |
| **miR-424-5p** | 0.3329 | 0.0358 | * |
| **miR-20a-5p** | -0.3319 | 0.0364 | * |
| **miR-103a-3p** | -0.3231 | 0.042 | * |
| **miR-30d-5p** | 0.3203 | 0.0439 | * |
| **miR-142-5p** | -0.32 | 0.0441 | * |
| **let-7i-5p** | 0.3165 | 0.0466 | * |
| **miR-23b-3p** | -0.3159 | 0.0471 | * |
|  |  |  | Continued on next page... |
|  |  |  |  |
|  |  |  |  |
|  |  |  |  |
|  |  |  |  |
| **% Excess weight lost** | | | |
| **MicroRNA** | **r** | **p** | **Significance** |
| **miR-99a-5p** | -0.4939 | 0.0012 | ** |
| **miR-148a-3p** | -0.4731 | 0.002 | ** |
| **miR-378a-3p** | -0.4564 | 0.0031 | ** |
| **miR-629-5p** | -0.4548 | 0.0032 | ** |
| **miR-320a** | -0.4439 | 0.0041 | ** |
| **miR-22-5p** | -0.4428 | 0.0042 | ** |
| **miR-301a-3p** | 0.4295 | 0.0057 | ** |
| **let-7d-3p** | -0.4277 | 0.0059 | ** |
| **miR-125b-5p** | -0.4235 | 0.0065 | ** |
| **miR-365a-3p** | -0.4165 | 0.0075 | ** |
| **let-7i-5p** | -0.4127 | 0.0081 | ** |
| **miR-374b-5p** | 0.4081 | 0.0089 | ** |
| **miR-660-5p** | -0.4026 | 0.01 | * |
| **miR-192-5p** | -0.3929 | 0.0121 | * |
| **miR-194-5p** | -0.369 | 0.0191 | * |
| **miR-423-3p** | -0.3669 | 0.0199 | * |
| **miR-148b-3p** | -0.3638 | 0.021 | * |
| **miR-92a-3p** | -0.3394 | 0.0322 | * |
| **miR-32-5p** | -0.3308 | 0.0371 | * |
| **miR-320b** | -0.3264 | 0.0399 | * |
| **miR-502-3p** | -0.3263 | 0.0399 | * |
| **miR-424-5p** | -0.3216 | 0.043 | * |
| **miR-486-5p** | -0.3174 | 0.0459 | * |
|  |  |  | Continued on next page... |
| **Fasting blood glucose** | | | |
| **MicroRNA** | **r** | **p** | **Significance** |
| **let-7b-3p** | 0.4951 | 0.0025 | ** |
| **miR-590-5p** | 0.4602 | 0.0054 | ** |
| **miR-30a-5p** | 0.4038 | 0.0162 | * |
| **miR-346** | -0.3917 | 0.02 | * |
| **miR-320a** | 0.3817 | 0.0237 | * |
| **miR-1** | 0.3594 | 0.034 | * |
| **miR-133a** | 0.356 | 0.0358 | * |
| **miR-30c-5p** | 0.3467 | 0.0413 | * |
| **miR-320b** | 0.3355 | 0.0488 | * |
|  |  |  |  |
| **Age** | | | |
| **MicroRNA** | **r** | **p** | **Significance** |
| **miR-136-5p** | 0.4572 | 0.0027 | ** |
| **miR-133a** | 0.4255 | 0.0055 | ** |
| **miR-301a-3p** | 0.4249 | 0.0056 | ** |
| **miR-20a-5p** | 0.3769 | 0.0152 | * |
| **miR-199a-5p** | 0.366 | 0.0186 | * |
| **miR-338-3p** | 0.3474 | 0.026 | * |
| **miR-23b-3p** | 0.3449 | 0.0272 | * |
| **miR-30c-5p** | 0.3418 | 0.0287 | * |
| **miR-30a-5p** | 0.3383 | 0.0305 | * |
| **miR-148a-3p** | -0.3371 | 0.0311 | * |
| **miR-148b-3p** | -0.3318 | 0.0341 | * |
| **miR-222-3p** | 0.3252 | 0.038 | * |
| **miR-144-5p** | 0.3209 | 0.0408 | * |
| **miR-1** | 0.3144 | 0.0453 | * |
| **miR-210** | 0.3131 | 0.0463 | * |
| **miR-152** | -0.3097 | 0.0488 | * |
| **miR-106a-5p** | 0.3094 | 0.049 | * |

^ⱡ^ Statistical significance: *p<0.05, **p<0.01, ***p<0.001.
